# Setting global research priorities for private sector child health service delivery: Results from a CHNRI exercise

**DOI:** 10.7189/jogh.10.021201

**Published:** 2020-12

**Authors:** Catherine Clarence, Tess Shiras, Jack Zhu, Malia K Boggs, Nefra Faltas, Anna Wadsworth, Sarah EK Bradley, Salim Sadruddin, Kerri Wazny, Catherine Goodman, Phyllis Awor, Zulfiqar A Bhutta, Karin Källander, Davidson H Hamer

**Affiliations:** 1 Abt Associates, International Development Division, Rockville, Maryland, USA; 2 Department of Global Health, Boston University School of Public Health, Boston, Massachusetts, USA; 3 United States Agency for International Development, Bureau for Global Health, Office of Maternal, Child Health and Nutrition, Washington, D.C., USA; 4 Save the Children, Washington, D.C., USA; 5 Johns Hopkins School of Public Health, Baltimore, Maryland, USA; 6 Department of Global Health and Development, London School of Hygiene and Tropical Medicine, London, UK; 7 Makerere University College of Health Sciences School of Public Health, Makerere, Uganda; 8 Centre for Global Child Health, The Hospital for Sick Children, Toronto, Canada; 9 Institute for Global Health & Development, The Aga Khan University, Karachi, Pakistan; 10 Implementation Research & Delivery Science Unit, UNICEF, New York, New York, USA; 11 Department of Global Public Health, Karolinska Institutet, Stockholm, Sweden; 12 Section of Infectious Diseases, Department of Medicine, Boston University School of Medicine, Boston, Massachusetts, USA

## Abstract

**Background:**

The private health sector is an important source of sick child care, yet evidence gaps persist in best practices for integrated management of private sector child health services. Further, there is no prioritized research agenda to address these gaps. We used a Child Health and Nutrition Research Initiative (CHNRI) process to identify priority research questions in response to these evidence gaps. CHNRI is a consultative approach that entails prioritizing research questions by evaluating them against standardized criteria.

**Methods:**

We engaged geographically and occupationally diverse experts in the private health sector and child health. Eighty-nine experts agreed to participate and provided 150 priority research questions. We consolidated submitted questions to reduce duplication into a final list of 50. We asked participants to complete an online survey to rank each question against 11 pre-determined criteria in four categories: (i) answerability, (ii) research feasibility, (iii) sustainability/equity, and (iv) importance/potential impact. Statistical data analysis was conducted in SAS 9.4 (SAS Institute Inc, Cary NC, USA). We weighted all 11 evaluation criteria equally to calculate the research priority score and average expert agreement for each question. We disaggregated results by location in high-income vs low- and middle-income countries.

**Results:**

Forty-nine participants (55.1%) completed the online survey, including 33 high-income and 16 low- and middle-income country respondents. The top, prioritized research question asks whether accreditation or regulation of private clinical and non-clinical sources of care would improve integrated management of childhood illness services. Four of the top ten research priorities were related to adherence to case management protocols. Other top research priorities were related to training and supportive supervision, digital health, and infant and newborn care. Research priorities among high-income and low- and middle-income country respondents were highly correlated.

**Conclusion:**

To our knowledge, this is the first systematic exercise conducted to define research priorities for the management of childhood illness in the private sector. The research priorities put forth in this CHNRI exercise aim to stimulate interest from policy makers, program managers, researchers, and donors to respond to and help close evidence gaps hindering the acceleration of reductions in child mortality through private sector approaches.

Since 1990, the global mortality rate among children under 5 years old has fallen by more than half [1]. Despite this significant reduction in child mortality, progress has been uneven. Accelerating reductions in child mortality and achieving the Sustainable Development Goals (SDGs) will require additional strategic investments and efforts across all health cadres and sectors, including the private sector. The private health sector is an important source of sick child care. A recent analysis of Demographic and Health Survey data from 24 low- and middle-income countries (LMICs) with a high child mortality burden, for example, revealed that on average 43% of caregivers in these countries sought care from the private sector when their child was sick with an acute respiratory infection (ARI), fever, or diarrhea [2].

However, effective approaches to harnessing and strengthening the private health sector to accelerate progress towards SDG targets for child health are not sufficiently documented or understood. A 2019 review of more than 1200 peer-reviewed and grey literature publications demonstrated that critical evidence gaps persist in best practices for child health programs implemented by the private health sector, particularly for integrated approaches, and that no prioritized research agenda exists to address these gaps [3].

Based on these findings, a core research team – composed of members from Boston University, the United States Agency for International Development’s (USAID's) flagship initiative in private sector health, Sustaining Health Outcomes through the Private Sector (SHOPS) Plus, and USAID – led a collaborative Child Health and Nutrition Research Initiative (CHNRI) process to identify priority research questions that would respond to major evidence gaps in private health sector approaches to case management of childhood illness. The private sector CHNRI focused on defining actionable research priorities that could result in private sector interventions and strategies to reduce morbidity and mortality among children under five, including newborns, in LMICs.

## What is CHNRI?

The CHNRI process is a consultative approach that entails identifying, compiling, and listing competing research questions, and then evaluating these research questions via a standardized set of criteria. The process engages experts from programmatic, research, donor, government, policy, and implementation backgrounds to participate in a six step process (see **Box 1**). CHNRI was originally developed in 2006 based on the recognized need to strengthen the global health evidence base, and on the need for a systematic approach to setting and making investment decisions on global child health and nutrition research priorities [4-6]. CHNRI has since become the most commonly used methodology for health research priority setting. The approach has been used for more than 50 health applications[7-9], including those related to integrated community case management (iCCM) of childhood illness [10], emerging interventions against childhood diarrhea [11], child protection in humanitarian settings [12], and pediatric and adolescent human immunodeficiency virus (HIV) [13]. There are many advantages to adopting this method to generate research priorities, including engaging a diverse group of relevant stakeholders; relying on a well-defined and systematic yet flexible process; and using a democratic, transparent approach to establishing a research agenda.

Box 1 **The CHNRI process entails six steps:**

1. Identify and invite experts to participate in the process

2. Determine criteria against which participants will evaluate all questions

3. Ask experts to submit priority research questions ideas (to be evaluated against criteria in step #2)

4. Consolidate and refine research questions to reduce duplication

5. Send prioritization survey to experts, asking them to evaluate submitted research questions

6. Analyze results

## Defining the private health sector

The private health sector is broad, and includes entities such as private health care providers, private insurance companies, and private actors across the health supply chain such as distributors and wholesalers of medical products. For the purposes of this CHNRI process, the private health sector is defined broadly to include for-profit providers, non-governmental organizations, social enterprises, marketing and franchising organizations, and faith-based organizations that deliver preventive and curative health services for children, particularly those under five. This includes both clinical and non-clinical private providers. Clinical providers are defined in this exercise to include those working in a clinic or other health facility setting. In contrast, non-clinical providers are those who work outside of a formal health facility setting, even if they have received some level of medical training. Examples of both clinical and non-clinical health providers are provided below.

- **Private clinical providers**: Doctors, nurses, and midwives at private primary, secondary, and tertiary level health facilities.
- **Private non-clinical providers**: Private pharmacists, drug shop workers, market sellers, and street vendors.

## Scope of the private sector CHNRI

This CHNRI focused on private health sector strategies related to the management of sick child care, with an emphasis on children under age 5. Recognizing that private health sector service delivery models may not fully align with integrated management of childhood illness (IMCI) protocols, the scope of this private sector CHNRI includes but also extends beyond the World Health Organization (WHO)-defined case management protocol for IMCI that is typically used in the public sector. We therefore encouraged participants in this CHNRI to interpret IMCI broadly, and to consider how IMCI or other integrated case management approaches could be adapted to private health sector models.

This article summarizes the results of our private health sector CHNRI and presents a prioritized research agenda for closing evidence gaps related to the management of sick child care through the private health sector. We expect that advancing this research will enhance the quality, efficiency, and sustainability of child health care services delivered by the private health sector, which will in turn contribute to meaningful global reductions in child morbidity and mortality.

## METHODS

The private sector CHNRI core research team adapted the CHNRI methodology for this study. The aim of using this methodology is to identify research gaps regarding the effectiveness of the private sector in improving the delivery and quality of health care interventions for children.

In addition to the core research team, a technical advisory group of six individuals provided substantial input throughout the CHNRI process. This technical advisory group provided technical input into defining the scope of the CHNRI exercise, identifying and finalizing the evaluation criteria, synthesizing and clarifying the list of submitted questions, and providing substantive feedback on the manuscript draft. Selected advisory group members also assisted with validation of analysis techniques.

In the following sections, we describe the methods for each of the six steps of the CHNRI process that are presented in **Box 1**.

### 1. Identify and invite experts to participate in the process

We proactively sought to engage a diverse group of individuals with expertise in the private health sector and child health to generate research questions. Using purposive and snowball sampling, these experts were drawn from the core research team’s global professional networks, referrals from individuals in these networks, members of the Private Sector Engagement and Implementation Science subgroups of the Child Health Task Force, and participants in the 2018 Global Symposium on Health Systems Research.

**Figure 1:**
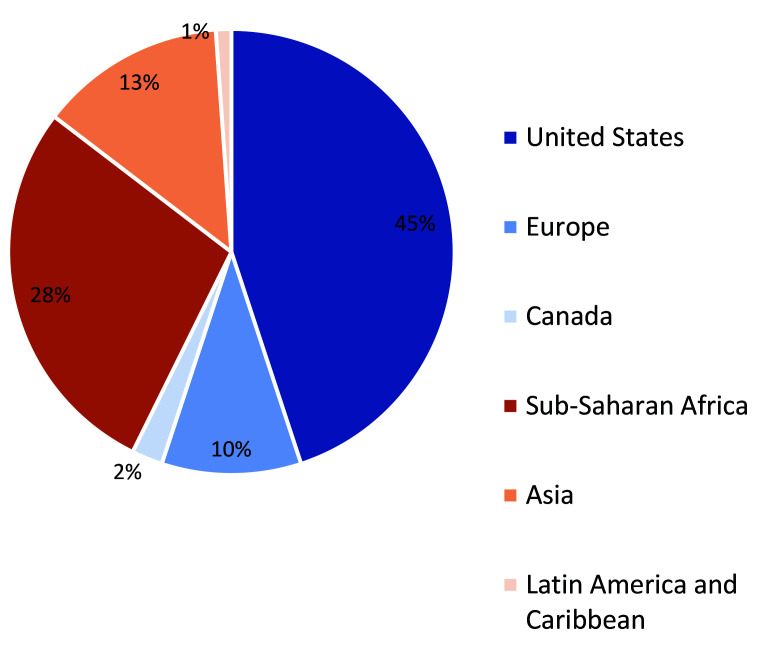
Distribution of CHNRI participants by geographic region. CHNRI − Child Health and Nutrition Research Initiative

**Figure 2:**
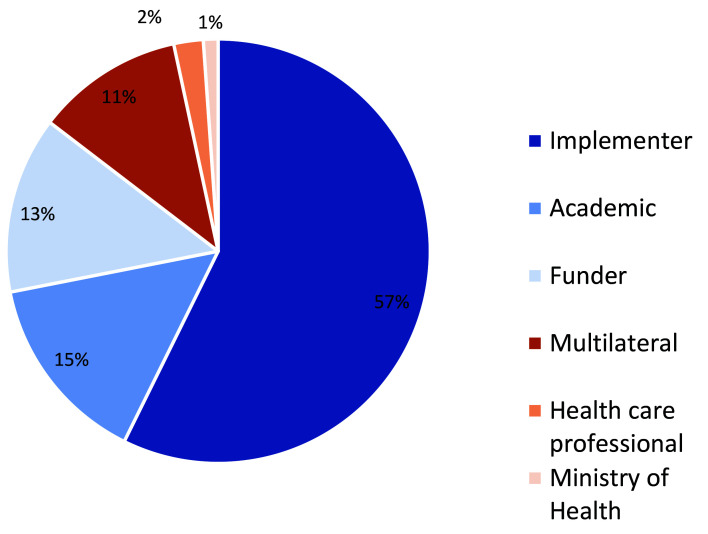
Distribution of CHNRI Participants. by type of institutional affiliation. CHNRI − Child Health and Nutrition Research Initiative.

In November 2018, we invited 129 technical experts to participate in the CHNRI exercise via email invitation. Nearly 90 global and country experts, representing academia, implementing partners, donors, bilateral organizations, and national and subnational Ministry of Health representatives, agreed to participate. **Figure 1** and **Figure 2** below illustrate the distribution of all CHNRI participants by geographic region and type of institutional affiliation, respectively.

### 2. Determine criteria against which participants will evaluate all questions

Next, the core research team determined the evaluation criteria that would be used to evaluate all proposed research questions submitted by respondents from among this group of global and country experts. We established four evaluation categories for proposed research questions: (i) answerability, (ii) research feasibility, (iii) sustainability/ equity, and (iv) importance/potential impact.

These evaluation criteria were adapted from those used during a previous CHNRI exercise conducted to establish a prioritized research agenda for iCCM [10]. Each of the four evaluation categories included multiple criteria, for a total of eleven evaluation criteria across the four evaluation categories (**Table 1**). The CHNRI expert group received these evaluation criteria and categories prior to being asked to submit their proposed research questions.

**Table 1:** Evaluation criteria for private sector CHNRI exercise

Category	Evaluation criteria
**Answerability**	1. Can a single study or a very small number of studies be designed to answer the research question?
	2. Does the research question have measurable outcome indicators?
**Research Feasibility**	3. Is it feasible to design and conduct a study in response to this research question? (*Considerations: potential time, cost, human resource needs, partnerships, technology, or training required to conduct the study*)
**Sustainability/Equity**	4. Depending upon the outcome of the research study, could this research result in a **sustainable** intervention or strategy to implement within the context of the private sector?
	5. Are the results from this research likely to result in a **scalable** intervention or strategy to implement through the private sector?
	6. Are the results from this research likely to lead to an intervention or strategy that will strengthen partnerships between the private sector and government?
	7. Will the results from this research lead to more equitable outcomes?
**Importance/Potential Impact**	8. Will the results of this research fill an important knowledge gap?
	9. Are the results from this research likely to inform future policy and practice?
	10. Will the results from this research be relevant to at least one aspect of the private sector across a range of low-and middle-income countries (as opposed to one country)?
	11. Will the results from the research help to strengthen quality of care provided by private health care providers (eg, clinicians, pharmacists, shop keepers)?

### 3. Ask experts to submit their ideas for priority research questions

We asked the 89 experts who agreed to participate in this CHNRI exercise in Step 1 to generate their own priority research questions. After receiving the evaluation criteria for proposed research questions that were established in Step 2, experts had one month to submit their ideas for priority research questions (from December 2018-January 2019). Over the course of this month, the core research team sent this expert group three email reminders to respond. Thirty-eight experts (43 percent) responded to this request, generating a total of nearly 150 ideas for priority research questions.

### 4. Consolidate and refine research questions to reduce duplication

Many of the research questions we received reflected similarities. This suggested some alignment of research priorities across expert respondents, and that the submitted questions had reached theoretical saturation. Under Step 4, we then consolidated and organized all submitted research question ideas, without altering the content of these ideas, to reduce redundancies and facilitate scoring and analysis. This consolidation exercise yielded a final list of 50 candidate research questions.

### 5. Send prioritization survey to experts, asking them to evaluate submitted research questions

In April 2019, we again contacted the 89 experts by email and asked them to evaluate the abovementioned list of 50 candidate research questions against the 11 evaluation criteria established under Step 2 of this CHNRI exercise (**Table 1**). Respondents were asked to share their feedback anonymously through an approximately 45-minute, online survey created using SurveyGizmo. In line with previous CHNRI applications [10,13], survey questions were listed in random order to reduce the possibility that question order would affect respondent scoring, and to mitigate differential respondent fatigue across questions. Respondents were asked to score each candidate research question using the CHNRI evaluation criteria they had received earlier, and according to a Likert scale ranging from 1 to 5 (with 1 = “No, strongly disagree,” and 5 = “Yes, strongly agree”). Respondents could also indicate if they were unable to evaluate a research question against a certain evaluation criterion by selecting the choice, “Do not know”. Respondents were given one month to complete the survey and received three email reminders from the core research team during this period.

### 6. Analyze results

We exported all survey responses from SurveyGizmo into an Excel spreadsheet. All 11 CHNRI evaluation criteria were determined to be of equal importance and were hence weighted equally, such that the sustainability/equity and importance/potential impact categories, of which included four evaluation criteria, had four times the weight of the research feasibility category, which included only one evaluation criterion, for example. The Research Priority Score (RPS), or average score across scorers and criteria, indicates the “collective optimism” among the scorers that a research question satisfies all 11 evaluation criteria [11]. The RPS was calculated separately for each proposed research question, summing the results of each of the 11 evaluation criteria questions, *q*, such that:


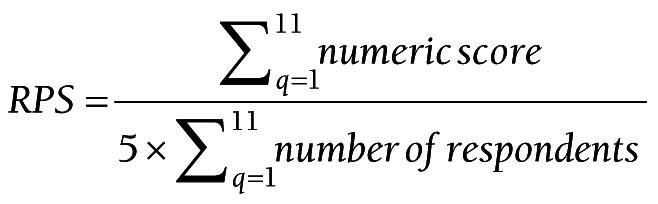


The numeric score for each criteria varied from 1 (strongly agree) to 5 (strongly disagree). Respondents who did not provide an answer for a particular evaluation criterion or who answered “do not know” were excluded from both the numerator and denominator.

We calculated the RPS separately for each proposed research question. Please see Figure S1 in the **Online Supplementary Document** for a more detailed version of the RPS calculation.

The evaluation criterion score for each of the eleven evaluation criteria was also calculated for each research question. This score is very similar to the RPS, but rather than an overall research question rating, indicates the collective optimism among the scorers that a research question satisfies a *particular evaluation criterion*. The evaluation criterion score was calculated for each research question and criterion as:


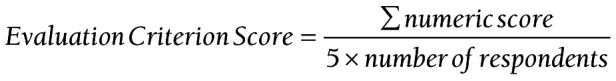


As noted above, respondents who did not provide an answer for a particular evaluation criterion or who answered “do not know” were excluded. Please see Figure S2 in the **Online Supplementary Document** for additional detail.

The Average Expert Agreement (AEA), the degree to which scorers were in consensus regarding the scores they provided, was also calculated. More specifically, the AEA describes the proportion of scorers who provided the mode, or most frequent Likert Scale score. For example, an AEA of 50 means that, on average, half of scorers agreed with one another and provided the same scores across all 11 evaluation criteria.

The AEA for each research question was calculated as:


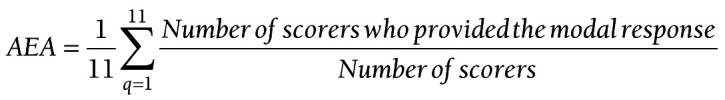


where q was the particular evaluation criterion that a research question was evaluated against.

Internet Protocol (IP) addresses at the time the respondent completed the online evaluation survey were used as a proxy measure for stratifying results and scores according to high-income country (HIC) and LMIC respondents and responses. Country classifications by income level were based on the 2019-2020 World Bank country classifications [14]. We calculated evaluation criteria scores, RPS, and AEAs according to these HIC and LMIC categories.

A Spearman’s rank correlation test was used to determine the correlation of the ranking of research questions between HICs and LMICs, as research priority ranking is an ordinal and non-parametric outcome. A Spearman’s Rho (ρ) or correlation ranges from -1 to 1, with 1 indicating a high, positive correlation between two groups and a -1 indicating a high negative correlation between two groups. A coefficient of 0 indicates no correlation.

Statistical data analysis was conducted in SAS 9.4 (IBM Inc, Armonk, NY, USA). We conducted two versions of this analysis: one version with completed surveys only and one version with completed surveys and four partially completed surveys that were at least 30% complete. The results of both types of analyses were very similar, so we chose to present only the analysis with complete surveys.

### Ethics statement

The Boston University Medical Campus Institutional Review Board reviewed this study and determined that it was not human subjects research [15].

## RESULTS

As aforementioned, 129 experts were invited to participate in the CHNRI exercise. Of those, 89 experts agreed to participate. Among the 89 participants, 38 (43 percent) submitted proposed research questions, and 49 (55 percent) completed online surveys in which they were asked to evaluate a consolidated list of 50 proposed research questions. Among the 49 respondents who completed the survey, 33 were located in HICs based on their IP address and 16 were located in LMICs.

The 15 overall, top-ranked research questions, evaluation criteria scores, RPS, interquartile ranges (IQR), and AEA are displayed in **Table 2**, and the data for all 50 questions are presented in Table S1 in the **Online Supplementary Document**.

**Table 2:** Overall rank, evaluation criteria scores, research priority scores, and average expert agreement for top 15 research questions among all respondents

Rank	1	2	3	4	5	6	7	8	9	10	11	12	13	14	15
Research question	Does accreditation or regulation of private clinical and non-clinical sources of care improve IMCI diagnosis, treatment, and appropriateness of testing and prescription?	Can supportive supervision lead to improved quality of care in the private sector?	What is the effectiveness of training private sector medicine vendors (ie, private drug shops, pharmacists, chemists, patent medicine vendors, etc.) to recognize, manage and/or refer sick young infants	Can tools (eg, flipchart, decision tree, and other job aids) used by private providers/pharmacies/drug shops improve adherence to child health protocols (diarrhea and pneumonia management, malaria treatment, and nutritional screening and counseling?	What are the key drivers of appropriate and inappropriate antimalarial and antibiotic prescription for children in private-for-profit sources of care by type of provider?	How can the integration of routine child health data from private sector providers (clinical and non-clinical) into national health information systems be improved and sustained?	What are the referral pathways in the private sector and what factors contribute to appropriate referrals to or from private sector providers?	What models of supportive supervision for child health service delivery are most cost-effective in the private sector?	What interventions are most effective in closing the gap between private provider knowledge and implementation of IMCI protocols?	What factors contribute to private provider adherence to IMCI protocols?	Can the iCCM approach be used in private non-clinical sources of care at scale to provide quality, appropriate, affordable, and accessible care?	Can government medicine regulatory authorities improve the quality of antimalarial medicines and antibiotics distributed by private drug shops or their equivalent through the use of periodic audits with a portable device to assess drug quality?	What can be done to reduce over-prescription of antibiotics when malaria rapid diagnostic testing results are negative and there are no other indications for antibiotic use?	What factors contribute to the gap between private provider knowledge of IMCI protocols and their implementation of IMCI protocols?	How well do private sector providers adhere to IMCI protocols?
**Evaluation criteria:**															
Answerability Question 1 Score: Single studies or small number of studies?	77	81	79	81	79	74	76	74	72	80	73	80	75	77	78
Answerability Question 2 Score: Measurable outcome indicators?	85	86	86	87	82	79	78	80	80	83	79	83	76	81	84
Research Feasibility Priority Score: Feasible to design and conduct study?	81	85	81	85	84	80	81	78	80	83	79	82	77	79	86
Sustainability and Equity Question 1 Score: Results in sustainable intervention/ strategy to implement within context of private sector?	84	83	81	83	81	82	79	81	82	80	78	78	78	78	70
Sustainability and Equity Question 2 Score: Results in scalable intervention/ strategy to implement within context of private sector?	86	79	78	83	79	79	77	80	78	77	78	76	77	75	71
Sustainability and Equity Question 3 Score: Results lead to intervention/strategy that strengthens partnerships between private sector and government?	83	78	77	68	74	82	80	76	75	71	74	72	72	73	70
Sustainability and Equity Question 4 Score: Results lead to more equitable outcomes?	75	71	77	68	70	70	73	71	73	69	78	71	69	70	71
Importance and Potential Impact Question 1 Score:Results fill an important knowledge gap?	81	80	80	74	82	85	83	83	81	80	79	78	81	78	77
Importance and Potential Impact Question 2 Score: Results inform future policy and practice?	84	83	83	78	79	83	81	82	82	79	82	78	81	78	78
Importance and Potential Impact Question 3 Score: Results relevant to at least one aspect of private sector across range of low- and middle-income countries?	83	83	82	81	78	84	80	82	81	80	80	80	81	79	78
Importance and Potential Impact Question 4 Score: Will the results from the research help to strengthen quality of care provided by private health providers	85	87	81	86	84	73	80	80	82	84	80	80	82	80	77
Research Priority Score (Interquartile Range)	82.1 (80.8-85.0)	81.5 (78.8-85.1)	80.3 (77.8-81.7)	79.6 (74.3-85.4)	79.3 (78.2-82.1)	79.3 (73.9-83.3)	79.0 (77.1-81.2)	78.9 (76.1-81.7)	78.8 (75.0-81.7)	78.6 (76.7-82.5)	78.2 (77.9-80.0)	78.0 (75.7-80.4)	77.3 (75.3-81.3)	77.1 (75.4-79.2)	76.5 (70.8-78.4)
Average Expert Agreement	52	47	40	51	48	47	43	49	53	48	43	51	47	47	42

IMCI – integrated management of childhood illness, iCCM − integrated community case management

The overall AEA ranged from 33 to 52 (out of 100). The mean AEA score for the top fifth of research priorities was 47.8 (IQR = 47-51), while the mean AEA for the bottom fifth of research priorities was 37.3 (IQR = 34-39).

The overall, highest-ranked priority research question regarding the management of childhood illness in the private sector asked whether the accreditation or regulation of private clinical and non-clinical sources of care would improve IMCI services (#1). The second ranking question asked if supportive supervision could lead to improved quality of care in the private sector (#2). Four of the top ten prioritized research questions were related to case management adherence (eg, can tools such a flip charts and decisions trees improve adherence to child health protocols in the private sector?). Other overall, top-ranked priority research questions were related to digital health, such as how the integration of routine child health data from private sector providers into national health information systems could be improved and sustained (#6), and infant/newborn care, such as the effectiveness of training private sector medicine vendors to recognize, manage and or refer sick young infants (#3).

The overall top ten research priorities are listed below from highest to lowest RPS:

1. Does accreditation or regulation of private clinical and non-clinical sources of care improve IMCI diagnosis, treatment, and appropriateness of testing and prescription? (RPS: 82.1)
2. Can supportive supervision lead to improved quality of care in the private sector? (RPS: 81.5)
3. What is the effectiveness of training private sector medicine vendors (i.e. private drug shops, pharmacists, chemists, patent medicine vendors, etc.) to recognize, manage and/or refer sick young infants? (RPS: 80.3)
4. Can tools (eg, flipchart, decision tree, and other job aids) used by private providers/pharmacies/drug shops improve adherence to child health protocols (diarrhea and pneumonia management, malaria treatment, and nutritional screening and counseling)? (RPS: 79.6)
5. What are the key drivers of appropriate and inappropriate antimalarial and antibiotic prescription for children in private-for-profit sources of care by type of provider? (RPS: 79.3)
6. How can the integration of routine child health data from private sector providers (clinical and non-clinical) into national health information systems be improved and sustained? (RPS: 79.3)
7. What are the referral pathways in the private sector and what factors contribute to appropriate referrals to or from private sector providers? (RPS: 79.0)
8. What models of supportive supervision for child health service delivery are most cost-effective in the private sector? (RPS: 78.9)
9. What interventions are most effective in closing the gap between private provider knowledge and implementation of IMCI protocols? (RPS: 78.8)
10. What factors contribute to private provider adherence to IMCI protocols? (RPS: 78.6)

### Stratified analysis by HIC and LMIC respondents

The evaluation results were very similar across respondents in HICs and LMICs. There was a strong and statistically significant correlation between the scores for HIC and LMIC respondents (Spearman’s *P* = 0.71, *P* < 0.0001), indicating that respondents from these varying locations largely prioritized similar research questions related to child health management in the private sector. **Table 3** and **Table 4**, respectively, show the top 10 research priorities as evaluated by HIC and LMIC participants. Questions on which both high-income and low-and middle-income country respondents agreed were among the top ten research priorities are highlighted in bold. For example, experts located in HICs and LMICs agreed that the following research questions were of utmost importance: what are the key drivers of appropriate and inappropriate antimalarial and antibiotic prescription for children in private-for-profit sources of care by type of provider (#6 among HIC respondents and #8 among LMIC respondents); can supportive supervision lead to improved quality of care in the private sector (#5 among HIC respondents and #1 among LMIC respondents); and what is the effectiveness of training private medicine vendors to recognize, manage, and/or refer sick young infants (#3 among HIC respondents and #10 among LMIC respondents). The complete list of research priorities as evaluated by HIC and LMIC participants is presented in Tables S2and S3 in the **Online Supplementary Document**, respectively.

**Table 3:** Rank, evaluation criteria scores, research priority scores, and average expert agreement for top ten questions in high income countries with complete surveys only (n = 18-33; varies by question)

Rank	1	2	3	4	5	6	7	8	9	10
Research question	Does accreditation or regulation of private clinical and non-clinical sources of care improve IMCI diagnosis, treatment, and appropriateness of testing and prescription?	Can tools (eg,flipchart, decision tree, and other job aids) used by private providers/pharmacies/drug shops improve adherence to child health protocols (diarrhea and pneumonia management, malaria treatment, and nutritional screening and counseling?	**What is the effectiveness of training private sector medicine vendors (ie, private drug shops, pharmacists, chemists, patent medicine vendors, etc.) to recognize, manage and/or refer sick young infants?**	Can government medicine regulatory authorities improve the quality of antimalarial medicines and antibiotics distributed by private drug shops or their equivalent through the use of periodic audits with a portable device to assess drug quality?	**Can supportive supervision lead to improved quality of care in the private sector?**	**What are the key drivers of appropriate and inappropriate antimalarial and antibiotic prescription for children in private-for-profit sources of care by type of provider?**	Can the iCCM approach be used in private non-clinical sources of care at scale to provide quality, appropriate, affordable, and accessible care?	What are the referral pathways in the private sector and what factors contribute to appropriate referrals to or from private sector providers?	What is the effect of social franchising with iCCM on access to child health care and outcomes?	**What factors contribute to private provider adherence to IMCI protocols?**
**Evaluation criteria:**											
Answerability Question 1 Score: Single studies or small number of studies?	76	79	78	79	76	75	69	73	70	77
Answerability Question 2 Score: Measurable outcome indicators?	86	87	85	86	84	81	78	76	77	82
Research Feasibility Priority Score: Feasible to design and conduct study?	80	86	79	83	83	82	78	78	75	80
Sustainability and Equity Question 1 Score: Results in sustainable intervention/ strategy to implement within context of private sector?	85	84	79	79	79	80	77	78	79	78
Sustainability and Equity Question 2 Score: Results in scalable intervention/ strategy to implement within context of private sector?	86	84	76	78	73	77	78	76	79	74
Sustainability and Equity Question 3 Score: Results lead to intervention/strategy that strengthens partnerships between private sector and government?	83	66	75	72	73	70	75	79	72	68
Sustainability and Equity Question 4 Score: Results lead to more equitable outcomes?	73	66	77	71	68	69	79	71	76	66
Importance and Potential Impact Question 1 Score:Results fill an important knowledge gap?	82	74	81	79	77	81	79	82	80	77
Importance and Potential Impact Question 2 Score: Results inform future policy and practice?	84	78	82	79	81	78	81	79	79	77
Importance and Potential Impact Question 3 Score: Results relevant to at least one aspect of private sector across range of low- and middle-income countries?	83	81	81	80	78	76	79	78	77	79
Importance and Potential Impact Question 4 Score: Will the results from the research help to strengthen quality of care provided by private health providers	85	88	80	82	85	85	79	78	75	83
Research Priority Score (interquartile range)	82.1 (80.0-85.0)	79.4 (74.2-85.6)	79.4 (76.7-81.3)	79.0 (78.1-81.9)	77.9 (73.1-82.6)	77.8 (74.8-81.3)	77.5 (76.9-79.4)	77.1 (75.8-78.8)	76.5 (75.3-79.4)	76.5 (74.4-80.0)
Average Expert Agreement	55	53	41	50	46	50	43	41	47	47

IMCI – integrated management of childhood illness, iCCM – integrated community case management

**Table 4:** Rank, evaluation criteria scores, research priority scores, and average expert agreement for top ten questions in low-and middle income countries with complete surveys only (n = 11-16; varies by question)

Rank	1	2	3	4	5	6	7	8	9	10
Research question	**Can supportive supervision lead to improved quality of care in the private sector?**	How can the integration of routine child health data from private sector providers (clinical and non-clinical) into national health information systems be improved and sustained?	What models of supportive supervision for child health service delivery are most cost-effective in the private sector?	What interventions are most effective in closing the gap between private provider knowledge and implementation of IMCI protocols?	What can be done to reduce over-prescription of antibiotics when malaria rapid diagnostic testing results are negative and there are no other indications for antibiotic use?	**What factors contribute to private provider adherence to IMCI protocols?**	What are the referral pathways in the private sector and what factors contribute to appropriate referrals to or from private sector providers?	**What are the key drivers of appropriate and inappropriate antimalarial and antibiotic prescription for children in private-for-profit sources of care by type of provider?**	How well do private sector providers adhere to IMCI protocols?	**What is the effectiveness of training private sector medicine vendors (ie, private drug shops, pharmacists, chemists, patent medicine vendors, etc.) to recognize, manage and/or refer sick young infants?**
**Evaluation criteria:**											
Answerability Question 1 Score: Single studies or small number of studies?	91	83	81	83	83	85	83	86	88	83
Answerability Question 2 Score: Measurable outcome indicators?	90	85	85	85	80	85	81	84	88	88
Research Feasibility Priority Score: Feasible to design and conduct study?	90	86	83	86	84	88	88	88	89	85
Sustainability and Equity Question 1 Score: Results in sustainable intervention/ strategy to implement within context of private sector?	90	88	88	87	83	84	80	83	78	84
Sustainability and Equity Question 2 Score: Results in scalable intervention/ strategy to implement within context of private sector?	90	86	86	86	84	81	80	83	75	81
Sustainability and Equity Question 3 Score: Results lead to intervention/strategy that strengthens partnerships between private sector and government?	89	89	87	81	79	75	84	81	76	80
Sustainability and Equity Question 4 Score: Results lead to more equitable outcomes?	76	76	76	75	76	74	76	72	76	78
Importance and Potential Impact Question 1 Score: Results fill an important knowledge gap?	88	89	85	83	88	85	85	83	83	76
Importance and Potential Impact Question 2 Score: Results inform future policy and practice?	88	89	86	85	85	84	85	81	85	84
Importance and Potential Impact Question 3 Score: Results relevant to at least one aspect of private sector across range of low- and middle-income countries?	91	90	81	85	88	84	84	83	84	84
Importance and Potential Impact Question 4 Score: Will the results from the research help to strengthen quality of care provided by private health providers	90	80	85	85	86	88	85	83	84	83
Research Priority Score (interquartile range)	88.5 (88.0-90.0)	85.5 (82.5-88.8)	83.9 (81.3-86.3)	83.7 (82.5-86.3)	83.2 (80.0-85.7)	82.9 (81.3-85.3)	82.7 (80.0-85.0)	82.4 (81.4-83.8)	82.2 (76.3-87.5)	82.2 (80.0-83.8)
Average Expert Agreement	54	51	52	52	45	53	50	44	45	43

IMCI – Integrated management of childhood illness

## DISCUSSION

To our knowledge, this is the first time the CHNRI approach has been used to define research gaps and priorities specific to the management of childhood illness in LMICs through *private sector* sources of care. The potential implications of this exercise are significant for many reasons: the crucial role of the private sector for management of sick children in many LMICs [2], the fact that many countries are not on track to meet SDG targets for reductions in under-five and neonatal mortality by 2030 [16], the paucity of literature available to understand the impact and sustainability potential for integrated case management of child health through the private sector, and the potential contributions of bridging these evidence gaps to accelerate reductions in under-five mortality. We expect the results of this CHNRI exercise will help shape the global research agenda for improving and expanding private health sector approaches to management of sick children.

The highest ranked research question asked whether accreditation or regulation of private clinical and non-clinical providers would improve appropriate testing, diagnosis, and treatment of sick children. This question scored second highest in terms of the ‘importance and potential impact to inform future policy and practice’ criterion, with an RPS of 84. However, the research question scored only 77 in terms of the first ‘answerability’ criterion, indicating the potential difficulty of designing a single study or a small number of studies to explore the question.

Across all HIC and LMIC respondents, experts agreed that research questions regarding case management adherence (#6 and 10 among HIC respondents and #6 and 8 among LMIC respondents) were of utmost importance. Of the top ten research questions, four were related to case management adherence in overall scoring, three were related to case management adherence when ranked just among HIC respondents, and five were related to case management adherence when ranked only among LMIC respondents. These questions included whether tools used by private providers can improve adherence to child health protocols (#4), understanding the key drivers of appropriate and inappropriate antimalarial and antibiotic prescription (#5), and what interventions are most effective in closing the gap between private provider knowledge and implementation of IMCI protocols (#9).

The third overall, highest ranked question asked about the effectiveness of training private sector medicine vendors to recognize, manage, and/or refer sick young infants (#3). This question also scored highly among the HIC- and LMIC-based respondents (#3 among HIC respondents and #10 among LMIC respondents). Addressing this question aligns with an area of critical need in advancing the global child health agenda, given that nearly half (47%) of all under-5 child deaths globally are among neonates [1].

Interestingly, while the research question (#6) on improving and sustaining the integration of routine child health data from private providers into national health information systems was *not* among HIC experts’ top ten research priorities, this question had the highest score across *all* expert respondents in terms of the ‘importance and potential impact to inform future policy and practice’ criterion. This is consistent with the fact that there has been increasing global attention to strengthening the integration of quality, routine child health data in national health information systems [17].

While perhaps not surprising, what is striking about a number of high-ranked research questions is how foundational they are – which reinforces the urgency to address them. A recent literature review of *Case Management of Childhood Illness in the Private Health Sector* summarized much of the current, available evidence base that can speak to some of these questions, highlighting that stark evidence gaps remain [3]. For example, a systematic review of iCCM interventions in Africa found that the majority of integrated case management interventions and evaluations, in fact, focus solely on malaria rather than on integrated care [18]. This review suggests opportunities to expand iCCM through private sector sources of care to improve equitable coverage of quality, lifesaving child health services [18]. A limited number of existing studies have also demonstrated that expanded provider knowledge across disease areas, including malaria, diarrhea, and pneumonia, can improve appropriate assessment of symptoms, reduce symptomatic dispensation of antimalarials, and improve case management and referrals for sick children. For example, an intervention in Nigeria found that using iCCM curricula led to marked success in improving the capacity of community-based patent medicine vendors to provide comprehensive and integrated treatment of childhood illnesses [19]. Several studies are also currently being implemented to answer a few of the high-ranking research questions from this exercise, including how to sustainably improve the routine reporting of data from private providers into national health information systems (ranked 6th overall), and improving appropriate referrals from private providers (ranked 7th).

Recently elevated global attention to certain technical areas may have influenced the respondents’ evaluation scores and overall ranks of research questions that reflect those global agendas. For example, many highly ranked research questions are related to quality of care, which has enjoyed significant visibility with the launch of the global Quality of Care Network in 2017 and release of new WHO *Standards for improving the quality of care for children and young adolescents in health facilities in 2018* [20]. Similarly, WHO and UNICEF released new guidelines in 2017 on *Operationalizing management of sick young infants with possible serious bacterial infection (PSBI) when referral is not feasible in the context of existing maternal, newborn, and child health programmes* [21], and recent years have seen a substantial increase in investments in and dissemination of implementation research on sick newborn and infant care. Since newborn deaths represent an increasing proportion of under-five deaths in many countries, this may also help explain the ranking of the research question on training private sector medicine vendors to recognize, manage, and refer sick young infants (#3) as third among HIC experts and tenth among LMIC experts. There have been several studies conducted in the last decade on community-level management of PSBI, and WHO released guidelines in 2015 for low resource settings in which referral for PSBI is not possible [22-24]. These studies have focused on the public sector, and there is a need to adapt best practices for the private sector. While Universal Health Coverage (UHC) is another topic that has seen high visibility with the creation of the global UHC2030 platform, proposed research questions pertaining to UHC did not rank among the overall top 15 across all respondents. For example, a question on opportunities and barriers for private clinical providers to support UHC through social health insurance schemes ranked 26th (out of 50) among all respondents. A question on the effect of iCCM in social franchises on access to child health services ranked ninth among respondents in HICs, but only 17th among all respondents.

Conversely, questions reflective of more nascent/emerging areas of research may have ranked lower in part *because* these are relatively underdeveloped areas of research with comparatively less global visibility. Such research questions arguably include those on using the private sector to improve coverage of child health services in hard-to-reach areas such as urban slums; understanding how the private sector can provide child health services in emergency settings; and understanding the role of private sector delivery of child health services in resilience building. Our findings on the low-ranked research question on child health in emergencies (#42) were also consistent with a 2018 adapted CHNRI exercise on global research priority setting in the overlapping field of child protection in humanitarian action, which did not include mention of the private sector among its top 15 global research priorities [12].

### Strengths and limitations

The CHNRI process can be a powerful tool to identify new research questions and to use input from a wide variety of stakeholders to define which questions should be prioritized. We used a similar approach to prior CHNRI exercises with some new twists. These included implementation through a web-based survey, randomization of research questions, an equal weighting system per question rather than category, and stratified analysis by participant location. In contrast to earlier CHNRI research question scoring activities, which were done in highly detailed Excel spreadsheets, we used a more user-friendly web-based approach. This strategy should be considered for future CHNRI research question priority activities. Randomization of research questions may help by reducing ‘question fatigue’ which can arise during the prolonged process of evaluating questions. Given the importance of each question, it seems more logical to give equal weight to the question rather than the overall category in which the question lies. Finally, stratifying the responses by participant location provides some insight into geographic differences, although this may be somewhat artificial since some respondents may be based in a specific location but actually represent a completely different part of the world.

While participation in this exercise included experts both from LMICs and HICs, we did face challenges with non-responsiveness to the CHNRI survey. The overall response rate for completed surveys was 55% and, as noted above, we included only fully completed surveys in our analysis. While lower than hoped, this response rate was similar to the response rate reported in the 2014 iCCM CHNRI [10]. Further, our sample size of 49 complete surveys aligns with previous CHNRI research demonstrating that collective opinion among experts stabilizes with a sample size of 45 to 55 experts [25]. There were also 62 partially completed surveys, many with just a question or two answered, and four of which were at least 30 percent complete. The high number of incomplete surveys may have been due in part to the ability of respondents to save, exit, and re-enter the online form multiple times, a design feature intended to encourage respondents to complete the entire survey if they could not do so in one sitting. Time to complete the survey may have also been a factor, as some respondents noted taking 1.5 to two hours to complete the online survey. Noting that different CHNRI exercises often adapt the number of evaluation criteria or even introduce new criteria to suit the needs of each exercise [7], future CHNRI exercises could include fewer evaluation criteria to reduce survey completion time and potential respondent fatigue However, doing so should be considered against the risk of reducing evaluation rigor.

Although we aimed to ‘crowd-source’ as many diverse experts with specialized knowledge in child health and private sector approaches, our targeting efforts did not reach all professionals working in this technical area. Future CHNRI endeavors could use a more extensive snowball sampling approach in which all CHNRI participants − not just the core research team, in the case of this CHNRI exercise − is asked to identify several additional participants. This kind of broader snowball sampling approach could further enhance the inclusiveness of the exercise, particularly among experts in LMICs. We also assumed that respondents were from a LMIC or HIC based on their IP address at the time of survey completion. While this was likely a reasonable assumption, it may not have been true for all experts – some of whom may have just been traveling to other regions at the time. To address this limitation, future CHNRI surveys could query respondents on their place of residence as a more direct (vs proxy) measure of their geographic designation as LMIC or HIC.

Several research questions may have been interpreted by survey respondents as overlapping in scope, which may have contributed to respondent fatigue, acquiescence, or confirmation bias. Additionally, as with any CHNRI exercise, there may have been other potential research investment options or ‘good ideas’ that were not proposed by our expert group and hence not included in the final list of research questions from which to choose (eg,no research questions were proposed related to private sector management of childhood malnutrition). Using the broader snowball sampling approach mentioned above to solicit ideas from a larger, more diverse group of participants may help mitigate this limitation for future CHNRI exercises. Finally, another limitation inherent to the CHNRI approach is that we do not have knowledge on how participants arrived at their submissions for priority research questions. The method relies on the expertise of the participants and does not ask participants to explain how or why they have selected their particular research questions. Future CHNRI iterations could ask participants to briefly explain the process they used to formulate their proposed research questions.

## CONCLUSION

The prioritized research agenda developed through this CHNRI exercise provides a foundation for intensified attention to and investment in research to advance evidence-based policies and practices for the management of childhood illness in the private sector. The fact that the CHNRI method was used for this priority-setting process is also significant, given what we feel is a critical need to ensure that global research agendas are established democratically, transparently, and with the collective ownership of the myriad stakeholders who will help drive their implementation. The research priorities put forth in this CHNRI exercise aim to stimulate interest and collaboration among policy makers, program managers, researchers, and donors to respond to and help close evidence gaps hindering the acceleration of reductions in child mortality through private sector approaches.

Much more could be done to harness the expertise and reach of private health sector providers to improve equitable access to lifesaving child health services and reduce millions of preventable childhood deaths each year. From the ongoing COVID-19 pandemic to Ebola, growing experience also suggests that total market approaches to delivering child and other health services that meaningfully engage both the public and private sectors may help build the resilience of health systems in the face of shocks and stresses [26,27]. We hope the results of this CHNRI exercise will contribute to global efforts to reposition the child health agenda to be more inclusive of private sector sources of care.

## Additional material

Online Supplementary Document
