# Supplementary material for: Setting global research priorities for private sector child health service delivery: Results from a CHNRI exercise
Source: J Glob Health. 2020 Dec 19;10(2):021201. doi: 10.7189/jogh.10.021201 (PMC7750021; doi:10.7189/jogh.10.021201)
Supplement: Online Supplementary Document [file jogh-10-021201-s001.pdf]

Supplementary Documents

**Figure S1: RPS Calculation**
The Research Priority Score (RPS), the average score across scorers and criteria, indicates the "collective optimism" among the scorers that a research question satisfies all 11 evaluation criteria (1).<br><br>The RPS was calculated separately for each proposed research question, summing the results of each of the 11 evaluation criteria questions, q, such that:<br><br>$RPS = \frac{\sum_{q=1}^{11} numeric\ score}{5*\sum_{q=1}^{11} number\ of\ respondents}$<br><br>The numeric score for each criteria varies from 1 (strongly agree) to 5 (strongly disagree).<br><br>A more detailed version of the RPS calculation is shown below for further clarity:<br><br>Let:<br>A1=answerability evaluation criteria 1<br>A2=answerability evaluation criteria 2<br>RF= research feasibility evaluation criteria<br>SE1= sustainability/equity evaluation criteria 1<br>SE2= sustainability/equity evaluation criteria 2<br>SE3=sustainability/equity evaluation criteria 3<br>SE4=sustainability/equity evaluation criteria 4<br>IP1=importance/potential impact evaluation criteria 1<br>IP2=importance/potential impact evaluation criteria 2<br>IP3=importance/potential impact evaluation criteria 3<br>IP4=importance/potential impact evaluation criteria 4*<br>*A "t" in front of the variable refers to the total score, while an "n" refers to the number of scorers for that variable<br><br>RPS= $\frac{\mathrm{tA1+tA2+tRF+tSE1+tSE2+tSE3+tSE4+tIP1+tIP2+tIP3+tIP4}}{5*(\mathrm{nA1+nA2+nRF+nSE1+nSE2+nSE3+nSE4+nIP1+nIP2+nIP3+nIP4})}$

**Figure S2: Evaluation Criteria Score Calculation**
The evaluation criterion score indicates the collective optimism among the scorers that a research question satisfies a particular evaluation criterion. The evaluation criterion score is calculated as follows, excluding from the numerator and denominator the respondents who did not score the particular evaluation criterion or who answered with “do not know”.<br><br>Evaluation Criteria Score: $\frac{\sum numeric\ score}{5*number\ of\ respondents}$

**Figure S3: AEA Calculation**
The Average Expert Agreement (AEA) is the degree to which scorers are in consensus regarding the scores they provided and is calculated as follows:<br><br>AEA= $\frac{1}{11}\sum_{q=1}^{11}\frac{Number\ of\ scorers\ who\ provided\ the\ modal\ response}{Number\ of\ scorers}$<br><br>where q is the particular evaluation criterion that a research question is being evaluated against. There are 11 evaluation criteria in total.

The first table (Table S1) shows the overall rank, evaluation criteria scores, Research Priority Scores, Interquartile Ranges (IQRs), and Average Expert Agreement (AEA) for all 50 research questions, from highest to lowest Research Priority Score when only the completed surveys are included in the analysis. The next two tables (Tables S2 and S3) show the rank, Research Priority Scores, IQRs and AEA for all 50 research questions, from highest to lowest Research Priority Score stratified by high income and low- and middle-income country respondent groups when only the complete surveys are included in the analysis.

Table S1: Overall rank, evaluation criteria scores, research priority scores, IQRs, and AEA for all 50 research questions with Complete Surveys Only

Overall Rank	Research Question (numbering refers to original number in questionnaire)	Evaluation Criteria											Research Priority Score (IQR)	Average Expert Agreement (AEA)
		Answerability Question 1 Score: Single studies or small number of studies?	Answerability Question 2 Score: Measurable outcome indicators?	Research Feasibility Priority Score: Feasible to design and conduct study?	Sustainability and Equity Question 1 Score: Results in sustainable intervention/ strategy to implement within context of private sector?	Sustainability and Equity Question 2 Score: Results in scalable intervention/strategy to implement within context of private sector?	Sustainability and Equity Question 3 Score: Results lead to intervention/ strategy that strengthens partnerships between private sector and government?	Sustainability and Equity Question 4 Score: Results lead to more equitable outcomes?	Importance and Potential Impact Question 1 Score: Results fill an important knowledge gap?	Importance and Potential Impact Question 2 Score: Results Inform future policy and practice?	Importance and Potential Impact Question 3 Score: Results relevant to at least one aspect of private sector across range of LMICs?	Importance and Potential Impact Question 4 Score		
1	Does accreditation or regulation of private clinical and non-clinical sources of care improve IMCI diagnosis, treatment, and appropriateness of testing and prescription?	77	85	81	84	86	83	75	81	84	83	85	82.1 (80.8-85.0)	52
2	Can supportive supervision lead to improved quality of care in the private sector?	81	86	85	83	79	78	71	80	83	83	87	81.5 (78.8-85.1)	47
3	What is the effectiveness of training private sector medicine vendors (i.e. private drug shops, pharmacists, chemists, patent medicine vendors, etc.) to recognize,	79	86	81	81	78	77	77	80	83	82	81	80.3 (77.8-81.7)	40

	manage and/or refer sick young infants?												
4	Can tools (e.g., flipchart, decision tree, and other job aids) used by private providers/pharmacies/drug shops improve adherence to child health protocols (diarrhea and pneumonia management, malaria treatment, and nutritional screening and counseling)?	81	87	85	83	83	68	68	74	78	81	86	79.6 (74.3-85.4)	51
5	What are the key drivers of appropriate and inappropriate antimalarial and antibiotic prescription for children in private-for-profit sources of care by type of provider?	79	82	84	81	79	74	70	82	79	78	84	79.3 (78.2-82.1)	48
6	How can the integration of routine child health data from private sector providers (clinical and non-clinical) into national health information systems be improved and sustained?	74	79	80	82	79	82	70	85	83	84	73	79.3 (73.9-83.3)	47
7	What are the referral pathways in the private sector and what factors contribute to appropriate referrals to or from private sector providers?	76	78	81	79	77	80	73	83	81	80	80	79.0 (77.1-81.2)	43

8	What models of supportive supervision for child health service delivery are most cost-effective in the private sector?	74	80	78	81	80	76	71	83	82	82	80	78.9 (76.1-81.7)	49
9	What interventions are most effective in closing the gap between private provider knowledge and implementation of IMCI protocols?	72	80	80	82	78	75	73	81	82	81	82	78.8 (75.0-81.7)	53
10	What factors contribute to private provider adherence to IMCI protocols?	80	83	83	80	77	71	69	80	79	80	84	78.6 (76.7-82.5)	48
11	Can the iCCM approach be used in private non-clinical sources of care at scale to provide quality, appropriate, affordable, and accessible care?	73	79	79	78	78	74	78	79	82	80	80	78.2 (77.9-80.0)	43
12	Can government medicine regulatory authorities improve the quality of antimalarial medicines and antibiotics distributed by private drug shops or their equivalent through the use of periodic audits with a portable device to assess drug quality?	80	83	82	78	76	72	71	78	78	80	80	78.0 (75.7-80.4)	51
13	What can be done to reduce over-prescription of antibiotics when malaria RDT results are negative and there are no other indications for antibiotic use?	75	76	77	78	77	72	69	81	81	81	82	77.3 (75.3-81.3)	47

14	What factors contribute to the gap between private provider knowledge of IMCI protocols and their implementation of IMCI protocols?	77	81	79	78	75	73	70	78	78	79	80	77.1 (75.4-79.2)	47
15	How well do private sector providers adhere to IMCI protocols?	78	84	86	70	71	70	71	77	78	78	77	76.5 (70.8-78.4)	42
16	What factors incentivize private sector providers to be accountable for high-quality IMCI implementation?	75	76	78	79	73	72	71	80	78	78	81	76.5 (73.5-78.8)	47
17	What is the effect of social franchising with iCCM on access to child health care and outcomes?	73	77	75	78	79	73	74	78	77	77	75	76.1 (74.1-77.8)	38
18	Can the private health sector improve the coverage of child health services among hard-to-reach populations, such as those living in rural areas and urban slums?	76	80	79	78	75	76	80	76	75	74	68	76.1 (74.7-79.1)	43
19	To what degree are private providers providing preventive services for children and how can they be incentivized to do so?	74	79	82	77	76	76	71	76	75	77	74	76.0 (73.9-77.0)	52
20	What are the most effective strategies for ensuring that private providers have access to national/government guidelines and training materials on the management of childhood illness?	76	73	76	78	76	80	69	69	77	79	76	75.5 (72.5-78.4)	46

21	What are the most cost-effective and efficient strategies to improve the capacity of private providers to deliver quality child health care?	65	75	73	78	77	70	70	80	79	76	77	74.5 (70.4-77.9)	50
22	Can the use of a text message (SMS) intervention lead to sustained improvements in the quality of iCCM delivered by private providers (clinical and non-clinical) for the management of malaria, pneumonia and diarrhea?	77	81	81	77	74	65	66	70	72	77	79	74.5 (69.8-78.8)	49
23	What are the cost and cost-effectiveness (e.g., in financial or other terms) of introducing IMCI/ICCM in the private sector? What is the impact on private provider revenue and profits?	74	80	78	75	74	71	66	79	77	75	70	74.5 (70.6-78.3)	47
24	What factors (internal and/or external) motivate care seeking for newborn and childhood illnesses from the private sector, for each provider cadre (e.g., private doctors, nurses, pharmacists, or shopkeepers)? *Note - internal factors include caregiver perceptions, social norms; external factors include cost, access.	75	80	80	76	72	68	69	75	76	76	70	74.4 (70.2-76.3)	50
25	What is the impact of introducing IMCI/iCCM in the private sector on private provider	78	85	83	72	69	69	63	77	73	77	67	73.9 (68.9-77.9)	43

	revenue and profits?													
26	What opportunities and barriers exist for private clinical providers to support achievement of universal health coverage for childhood illnesses through participation in social health insurance schemes?	69	70	73	75	73	76	75	76	76	78	69	73.5 (70.2-75.7)	37
27	Given the prevalence of non-clinical providers in many countries, what types of models/incentives work to formalize/integrate private non-clinical providers into the health system in a sustainable manner?	64	68	70	75	74	79	73	79	77	76	75	73.5 (69.8-76.7)	39
28	What is the impact of adding services, such as iCCM or IMCI, to various socially-franchised outlets? Is there an optimum package that can be added without compromising the quality of existing services?	71	72	74	77	74	67	68	75	76	78	74	73.4 (71.3-75.7)	44
29	Could private sector IMCI delivery be improved using digital health applications and/or big data?	72	72	75	76	74	72	64	73	72	80	76	73.3 (72.2-75.7)	48
30	Do private health providers (clinical and nonclinical) promote infant formula rather than exclusive	74	83	81	71	69	65	64	71	73	72	74	72.7 (68.5-74.3)	36

	breastfeeding and, if so, why?													
31	What institutional arrangements, regulatory and otherwise, would allow private providers to be systematically integrated into national child health programs in a sustainable manner?	65	65	70	76	73	77	71	75	78	76	71	72.6 (70.4-76.3)	45
32	How can logistics management information systems be used to track and prevent stock-outs of essential childhood medicines within private sector sources of care?	70	75	74	73	73	72	67	71	71	75	73	72.2 (70.9-73.6)	45
33	What are the beneficial and negative effects of entrepreneurial models on private community health provider performance and on child health outcomes?	65	71	74	75	72	69	70	76	72	76	71	71.9 (70.0-74.8)	36
34	How can the skills of private providers in implementing IMCI protocols be sustained?	66	70	71	75	70	68	66	78	76	75	75	71.7 (67.5-75.0)	43
35	Are private providers receiving appropriate training and, if so, are they providing a quality of child care that is commensurate with their training?	68	72	74	69	69	67	65	72	72	73	73	70.4 (67.9-72.9)	44
36	What are the key factors to developing and implementing large-scale, sustainable public-	61	63	64	74	72	76	67	75	78	79	67	70.4 (63.6-75.7)	41

	private-partnerships, or public-private collaboration to deliver child health care (taking into account financing, supply chain, regulation, and building trust between public and private sectors)?													
37	How can countries harness the existing supply systems for other consumer goods and commodities to deliver IMCI commodities in hard-to-reach private health facilities?	68	69	70	73	69	70	67	73	70	74	70	70.3 (69.3-73.2)	39
38	What are the key health system components needed to implement guidelines on outpatient management of young infants with possible severe bacterial infection in the private sector?	69	69	69	70	66	70	63	73	73	75	74	70.1 (68.6-73.3)	40
39	Do private sector providers provide greater access to child health services for illnesses/conditions that are underserved by public sector sources of care?	73	77	72	68	65	68	70	71	74	72	61	70.0 (68.1-72.8)	40
40	Is it feasible to use an external quality assurance mechanism for malaria RDT quality control based on nucleic acid amplification techniques in private non-clinical sources of care?	77	77	74	68	67	63	60	65	71	74	69	69.4 (64.8-74.2)	45

41	How can private supply chain systems (e.g., Pharmaceutical companies, manufacturers, whole-salers, importers) be incentivized to provide quality products?	65	69	67	70	68	65	64	71	69	75	71	68.6 (65.1-70.9)	40
42	How can the private sector provide services in emergency settings?	65	64	66	69	64	70	70	73	74	73	64	68.3 (64.0-72.6)	39
43	How can private supply chain systems (e.g., Pharmaceutical companies, manufacturers, whole-salers, importers) be incentivized to ensure private providers do not face stock-outs?	66	74	68	70	66	63	65	69	67	73	68	68.0 (66.1-70.0)	37
44	How can non-governmental regulation (e.g., from oneself, one's peers, clients, the community) of private providers improve quality assurance in the private health sector?	66	68	69	68	64	62	64	70	70	73	72	67.9 (64.3-70.2)	39
45	How much value do private providers place on government training, government-approved training materials, and assessment and treatment protocols?	73	70	76	63	60	69	54	64	69	72	66	66.8 (62.9-71.7)	44
46	What is the role of the private sector in care seeking for children five years and older (up to 18 years old)?	65	70	73	64	61	64	60	71	67	70	60	65.9 (61.3-69.8)	35

47	What are the differences in health outcomes between children receiving care from the private sector and public sectors?	63	76	65	61	58	58	60	71	67	69	65	64.9 (60.0-69.1)	34
48	Can private sector delivery of child health services contribute to more resilient households, communities, and/or health systems, and if so, how can this be effectively measured?	54	54	55	62	59	61	64	69	63	69	59	60.8 (55.0-64.3)	38
49	How can pharmaceutical companies be incentivized to transfer knowledge nationwide regarding appropriate care for sick children?	58	58	58	62	59	57	58	63	62	68	64	60.6 (58.2-62.7)	34
50	Are there unintended negative consequences of implementing IMCI in the private sector?	60	60	60	59	57	54	56	65	63	65	64	60.2 (56.6-63.8)	33

Table S2: Rank, Research Priority Scores, IQRs, and Average Expert Agreement for All Fifty Questions from High Income Country Respondents with Complete Surveys Only			
Overall Rank	Research Question (numbering refers to original number in questionnaire)	Research Priority Score (IQR)	Average Expert Agreement (AEA)
1	Does accreditation or regulation of private clinical and non-clinical sources of care improve IMCI diagnosis, treatment, and appropriateness of testing and prescription?	82.1 (80.0-85.0)	55
2	Can tools (e.g., flipchart, decision tree, and other job aids) used by private providers/pharmacies/drug shops improve adherence to child health protocols (diarrhea and pneumonia management, malaria treatment, and nutritional screening and counseling)?	79.4 (74.2-85.6)	53
3	What is the effectiveness of training private sector medicine vendors (i.e. private drug shops, pharmacists, chemists, patent medicine vendors, etc.) to recognize, manage and/or refer sick young infants?	79.4 (76.7-81.3)	41
4	Can government medicine regulatory authorities improve the quality of antimalarial medicines and antibiotics distributed by private drug shops or their equivalent through the use of periodic audits with a portable device to assess drug quality?	79.0 (78.1-81.9)	50
5	Can supportive supervision lead to improved quality of care in the private sector?	77.9 (73.1-82.6)	46
6	What are the key drivers of appropriate and inappropriate antimalarial and antibiotic prescription for children in private-for-profit sources of care by type of provider?	77.8 (74.8-81.3)	50
7	Can the iCCM approach be used in private non-clinical sources of care at scale to provide quality, appropriate, affordable, and accessible care?	77.5 (76.9-79.4)	43
8	What are the referral pathways in the private sector and what factors	77.1 (75.8-78.8)	41

	contribute to appropriate referrals to or from private sector providers?		
9	What is the effect of social franchising with iCCM on access to child health care and outcomes?	76.5 (75.3-79.4)	47
10	What factors contribute to private provider adherence to IMCI protocols?	76.5 (74.4-80.0)	47
11	What interventions are most effective in closing the gap between private provider knowledge and implementation of IMCI protocols?	76.4 (72.7-80.0)	53
12	What models of supportive supervision for child health service delivery are most cost-effective in the private sector?	76.4 (71.0-79.4)	49
13	How can the integration of routine child health data from private sector providers (clinical and non-clinical) into national health information systems be improved and sustained?	76.2 (69.7-80.6)	49
14	To what degree are private providers providing preventive services for children and how can they be incentivized to do so?	76.1 (74.4-78.7)	54
15	What are the most effective strategies for ensuring that private providers have access to national/government guidelines and training materials on the management of childhood illness?	75.9 (73.1-78.2)	49
16	Can the private health sector improve the coverage of child health services among hard-to-reach populations, such as those living in rural areas and urban slums?	75.1 (73.1-78.1)	46
17	What factors contribute to the gap between private provider knowledge of IMCI protocols and their implementation of IMCI protocols?	75.0 (71.5-77.0)	47
18	What is the impact of adding services, such as iCCM or IMCI, to various socially-franchised outlets? Is there an optimum package that can be added without compromising the quality of existing services?	74.9 (71.0-78.8)	46

19	What can be done to reduce over-prescription of antibiotics when malaria RDT results are negative and there are no other indications for antibiotic use?	74.7 (71.9-78.7)	51
20	What factors incentivize private sector providers to be accountable for high-quality IMCI implementation?	74.6 (70.3-77.5)	50
21	How well do private sector providers adhere to IMCI protocols?	73.8 (68.7-74.8)	44
22	Can the use of a text message (SMS) intervention lead to sustained improvements in the quality of iCCM delivered by private providers (clinical and non-clinical) for the management of malaria, pneumonia and diarrhea?	73.6 (71.3-79.4)	52
23	What factors (internal and/or external) motivate care seeking for newborn and childhood illnesses from the private sector, for each provider cadre (e.g., private doctors, nurses, pharmacists, or shopkeepers)? *Note - internal factors include caregiver perceptions, social norms; external factors include cost, access.	73.1 (69.7-75.2)	52
24	What are the cost and cost-effectiveness (e.g., in financial or other terms) of introducing IMCI/ICCM in the private sector? What is the impact on private provider revenue and profits?	72.4 (68.8-76.3)	49
25	How can logistics management information systems be used to track and prevent stock-outs of essential childhood medicines within private sector sources of care?	72.4 (70.6-74.2)	46
26	What are the beneficial and negative effects of entrepreneurial models on private community health provider performance and on child health outcomes?	72.4 (71.0-75.7)	37
27	What is the impact of introducing IMCI/iCCM in the private sector on private provider revenue and profits?	71.8 (66.0-75.0)	45

28	Given the prevalence of non-clinical providers in many countries, what types of models/incentives work to formalize/integrate private non-clinical providers into the health system in a sustainable manner?	71.7 (68.4-75.8)	41
29	What are the most cost-effective and efficient strategies to improve the capacity of private providers to deliver quality child health care?	71.6 (68.0-75.6)	47
30	Is it feasible to use an external quality assurance mechanism for malaria RDT quality control based on nucleic acid amplification techniques in private non-clinical sources of care?	71.3 (66.7-77.0)	47
31	Could private sector IMCI delivery be improved using digital health applications and/or big data?	71.2 (68.0-72.9)	46
32	What institutional arrangements, regulatory and otherwise, would allow private providers to be systematically integrated into national child health programs in a sustainable manner?	71.1 (68.8-75.6)	50
33	What opportunities and barriers exist for private clinical providers to support achievement of universal health coverage for childhood illnesses through participation in social health insurance schemes?	70.6 (66.7-74.0)	39
34	Do private health providers (clinical and nonclinical) promote infant formula rather than exclusive breastfeeding and, if so, why?	70.4 (66.5-73.1)	37
35	Do private sector providers provide greater access to child health services for illnesses/conditions that are underserved by public sector sources of care?	70.3 (67.1-74.7)	43
36	What are the key factors to developing and implementing large-scale, sustainable public-private-partnerships, or public-private collaboration to deliver child health care (taking into account financing, supply chain, regulation, and building	70.3 (60.7-76.6)	39

	trust between public and private sectors)?		
37	How can the skills of private providers in implementing IMCI protocols be sustained?	69.9 (65.6-74.4)	42
38	How can countries harness the existing supply systems for other consumer goods and commodities to deliver IMCI commodities in hard-to-reach private health facilities?	69.6 (67.7-73.3)	40
39	How can private supply chain systems (e.g., Pharmaceutical companies, manufacturers, whole-salers, importers) be incentivized to ensure private providers do not face stock-outs?	69.4 (65.6-72.9)	37
40	What are the key health system components needed to implement guidelines on outpatient management of young infants with possible severe bacterial infection in the private sector?	69.0 (66.7-72.5)	42
41	How can the private sector provide services in emergency settings?	68.0 (62.1-72.7)	41
42	How can private supply chain systems (e.g., Pharmaceutical companies, manufacturers, whole-salers, importers) be incentivized to provide quality products?	67.9 (65.3-70.0)	40
43	How much value do private providers place on government training, government-approved training materials, and assessment and treatment protocols?	65.6 (59.4-71.3)	48
44	Are private providers receiving appropriate training and, if so, are they providing a quality of child care that is commensurate with their training?	65.5 (62.0-67.7)	38
45	How can non-governmental regulation (e.g., from oneself, one's peers, clients, the community) of private providers improve quality assurance in the private health sector?	65.4 (62.5-68.0)	34

46	What is the role of the private sector in care seeking for children five years and older (up to 18 years old)?	62.7 (58.1-65.6)	37
47	What are the differences in health outcomes between children receiving care from the private sector and public sectors?	61.3 (55.9-68.0)	34
48	How can pharmaceutical companies be incentivized to transfer knowledge nationwide regarding appropriate care for sick children?	60.6 (58.1-62.6)	33
49	Can private sector delivery of child health services contribute to more resilient households, communities, and/or health systems, and if so, how can this be effectively measured?	59.3 (51.4-63.3)	39
50	Are there unintended negative consequences of implementing IMCI in the private sector?	57.3 (54.4-61.3)	33

**Table S3: Rank, Research Priority Scores, IQRs, and Average Expert Agreement for All Fifty Questions from Low-and Middle Income Country respondents with Complete Surveys Only**			
Overall Rank	Research Question (numbering refers to original number in questionnaire)	Research Priority Score (IQR)	Average Expert Agreement (AEA)
1	Can supportive supervision lead to improved quality of care in the private sector?	88.5 (88.0-90.0)	54
2	How can the integration of routine child health data from private sector providers (clinical and non-clinical) into national health information systems be improved and sustained?	85.5 (82.5-88.8)	51
3	What models of supportive supervision for child health service delivery are most cost-effective in the private sector?	83.9 (81.3-86.3)	52
4	What interventions are most effective in closing the gap between private provider knowledge and implementation of IMCI protocols?	83.7 (82.5-86.3)	52
5	What can be done to reduce over-prescription of antibiotics when malaria RDT results are negative and there are no other indications for antibiotic use?	83.2 (80.0-85.7)	45
6	What factors contribute to private provider adherence to IMCI protocols?	82.9 (81.3-85.3)	53
7	What are the referral pathways in the private sector and what factors contribute to appropriate referrals to or from private sector providers?	82.7 (80.0-85.0)	50
8	What are the key drivers of appropriate and inappropriate antimalarial and antibiotic prescription for children in private-for-profit sources of care by type of provider?	82.4 (81.4-83.8)	44
9	How well do private sector providers adhere to IMCI protocols?	82.2 (76.3-87.5)	45
10	What is the effectiveness of training private sector medicine vendors (i.e. private drug shops, pharmacists, chemists, patent medicine vendors, etc.) to recognize, manage and/or refer sick young infants?	82.2 (80.0-83.8)	43
11	Does accreditation or regulation of private clinical and non-clinical sources of care improve IMCI diagnosis, treatment, and appropriateness of testing and prescription?	82.2 (80.0-85.0)	48
12	What factors contribute to the gap between private provider knowledge of IMCI protocols and their implementation of IMCI protocols?	81.3 (80.0-84.0)	50
13	What factors incentivize private sector providers to be accountable for high-quality IMCI implementation?	80.5 (80.0-82.5)	45
14	What are the most cost-effective and efficient strategies to improve the capacity of private providers to deliver quality child health care?	80.2 (76.3-82.7)	58
15	Are private providers receiving appropriate training and, if so, are they providing a quality of child care that is commensurate with their training?	80.0 (77.5-83.8)	58

16	Can tools (e.g., flipchart, decision tree, and other job aids) used by private providers/pharmacies/drug shops improve adherence to child health protocols (diarrhea and pneumonia management, malaria treatment, and nutritional screening and counseling)?	79.9 (74.7-85.0)	53
17	Can the iCCM approach be used in private non-clinical sources of care at scale to provide quality, appropriate, affordable, and accessible care?	79.7 (78.7-81.3)	43
18	What opportunities and barriers exist for private clinical providers to support achievement of universal health coverage for childhood illnesses through participation in social health insurance schemes?	79.4 (78.7-80.0)	44
19	What are the cost and cost-effectiveness (e.g., in financial or other terms) of introducing IMCI/ICCM in the private sector? What is the impact on private provider revenue and profits?	79.0 (74.7-82.9)	44
20	Can the private health sector improve the coverage of child health services among hard-to-reach populations, such as those living in rural areas and urban slums?	78.2 (75.0-81.3)	42
21	What is the impact of introducing IMCI/iCCM in the private sector on private provider revenue and profits?	78.2 (74.7-84.0)	40
22	Could private sector IMCI delivery be improved using digital health applications and/or big data?	77.5 (76.0-81.3)	54
23	Do private health providers (clinical and nonclinical) promote infant formula rather than exclusive breastfeeding and, if so, why?	77.1 (72.5-83.8)	39
24	Given the prevalence of non-clinical providers in many countries, what types of models/incentives work to formalize/integrate private non-clinical providers into the health system in a sustainable manner?	77.0 (75.0-78.8)	40
25	What factors (internal and/or external) motivate care seeking for newborn and childhood illnesses from the private sector, for each provider cadre (e.g., private doctors, nurses, pharmacists, or shopkeepers)? *Note - internal factors include caregiver perceptions, social norms; external factors include cost, access.	76.9 (72.5-80.0)	49
26	Can the use of a text message (SMS) intervention lead to sustained improvements in the quality of iCCM delivered by private providers (clinical and non-clinical) for the management of malaria, pneumonia and diarrhea?	76.3 (70.0-81.3)	47
27	Can government medicine regulatory authorities improve the quality of antimalarial medicines and antibiotics distributed by private drug shops or their equivalent through	76.0 (73.3-78.7)	57

	the use of periodic audits with a portable device to assess drug quality?		
28	To what degree are private providers providing preventive services for children and how can they be incentivized to do so?	75.7 (72.5-78.8)	47
29	What institutional arrangements, regulatory and otherwise, would allow private providers to be systematically integrated into national child health programs in a sustainable manner?	75.5 (72.5-77.3)	38
30	What is the effect of social franchising with iCCM on access to child health care and outcomes?	75.4 (73.8-77.3)	41
31	How can the skills of private providers in implementing IMCI protocols be sustained?	75.2 (73.8-77.5)	47
32	What are the most effective strategies for ensuring that private providers have access to national/government guidelines and training materials on the management of childhood illness?	74.7 (71.3-77.5)	42
33	How can non-governmental regulation (e.g., from oneself, one's peers, clients, the community) of private providers improve quality assurance in the private health sector?	72.7 (68.8-76.3)	55
34	What is the role of the private sector in care seeking for children five years and older (up to 18 years old)	72.4 (68.0-78.7)	35
35	What are the key health system components needed to implement guidelines on outpatient management of young infants with possible severe bacterial infection in the private sector?	72.3 (68.0-77.1)	37
36	What are the differences in health outcomes between children receiving care from the private sector and public sectors?	72.2 (70.0-75.0)	42
37	How can logistics management information systems be used to track and prevent stock-outs of essential childhood medicines within private sector sources of care?	72.0 (69.3-74.7)	44
38	How can countries harness the existing supply systems for other consumer goods and commodities to deliver IMCI commodities in hard-to-reach private health facilities?	71.7 (69.3-74.3)	40
39	What are the beneficial and negative effects of entrepreneurial models on private community health provider performance and on child health outcomes?	71.0 (70.0-72.5)	39
40	What are the key factors to developing and implementing large-scale, sustainable public-private-partnerships, or public-private collaboration to deliver child health care	70.5 (67.5-73.8)	49

	(taking into account financing, supply chain, regulation, and building trust between public and private sectors)?		
41	What is the impact of adding services, such as iCCM or IMCI, to various socially-franchised outlets? Is there an optimum package that can be added without compromising the quality of existing services?	70.2 (68.0-74.7)	43
42	How can private supply chain systems (e.g., Pharmaceutical companies, manufacturers, whole-salers, importers) be incentivized to provide quality products?	69.8 (65.0-72.5)	51
43	Do private sector providers provide greater access to child health services for illnesses/conditions that are underserved by public sector sources of care?	69.3 (66.7-73.3)	37
44	How much value do private providers place on government training, government-approved training materials, and assessment and treatment protocols?	69.2 (67.5-71.3)	38
45	How can the private sector provide services in emergency settings?	69.0 (66.3-71.3)	43
46	Are there unintended negative consequences of implementing IMCI in the private sector?	66.1 (61.3-69.3)	39
47	Is it feasible to use an external quality assurance mechanism for malaria RDT quality control based on nucleic acid amplification techniques in private non-clinical sources of care?	66.0 (61.8-69.1)	49
48	How can private supply chain systems (e.g., Pharmaceutical companies, manufacturers, whole-salers, importers) be incentivized to ensure private providers do not face stock-outs?	65.2 (62.7-68.0)	42
49	Can private sector delivery of child health services contribute to more resilient households, communities, and/or health systems, and if so, how can this be effectively measured?	63.6 (61.3-66.3)	43
50	How can pharmaceutical companies be incentivized to transfer knowledge nationwide regarding appropriate care for sick children?	60.7 (56.3-62.5)	50

References

1. Bhutta ZA, Zipursky A, Wazny K, Levine MM, Black RE, Bassani DG, et al. Setting priorities for development of emerging interventions against childhood diarrhoea. J Glob Health [Internet]. 2013 Jun [cited 2020 Jan 29];3(1). Available from: https://www.ncbi.nlm.nih.gov/pmc/articles/PMC3700035/
